# Dolutegravir developmental toxicity is mitigated by magnesium and folate in zebrafish embryos

**DOI:** 10.1242/dmm.052632

**Published:** 2026-05-26

**Authors:** Robert M. Cabrera, Ahmed Mohamed, Ryoko Minowa, Katheryn A. Neugebauer, Daniel A. Gorelick

**Affiliations:** Center for Precision Environmental Health, Department of Molecular and Cellular Biology, Baylor College of Medicine, Houston, TX 77030, USA

**Keywords:** Zebrafish, Birth defects, Drug toxicity, Magnesium, Folate

## Abstract

Integrase strand transfer inhibitors have transformed human immunodeficiency virus (HIV) therapy, yet the widely prescribed drug dolutegravir (DTG) has been linked to developmental toxicity, and its teratogenic mechanism remains unclear. Here, we used zebrafish to dissect DTG toxicity during early vertebrate development. DTG exposure from 2-4 h post-fertilization (hpf) to 24 hpf produced high mortality and abnormal morphology. Co-treatment with folates partially restored normal morphology, whereas calcium had no effect. Strikingly, supplementation with magnesium (Mg) partially rescued DTG-exposed embryos, implicating Mg availability in protection. In competitive binding assays, Mg increased binding of folate to purified folate receptor (FOLR1) by 30% in the presence of DTG. *folr1* mutant embryos contained significantly less endogenous folate than wild-type embryos and displayed marked hypersensitivity to DTG that could not be mitigated by folate supplementation. Critically, Mg supplementation partially rescued DTG toxicity in *folr1* mutants, indicating a Folr1-independent component and placing the balance between free DTG and Mg-bound DTG upstream of folate transport. These results support a model in which free DTG antagonizes FOLR1 and Mg modifies DTG developmental toxicity through FOLR1-dependent and -independent processes.

## Teaser

**Editor's choice:** Dolutegravir disrupts folate signaling during early development, causing embryonic defects that are prevented by folate or magnesium, revealing a mechanistic link between integrase inhibitors, folate biology and developmental toxicity.

## INTRODUCTION

Dolutegravir (DTG) is an effective and preferred antiretroviral therapy worldwide owing to its potency, high genetic barrier to resistance and convenient once-daily dosing. In 2018, however, nationwide surveillance in Botswana reported higher prevalence of neural-tube defects (NTDs) among infants whose mothers conceived while taking DTG than among those receiving other regimens or no antiretrovirals (Zash et al., 2018, 2019). More recent analyses have tempered that concern. An Eswatini cohort, conducted in a setting that, like Botswana, lacks mandatory folic acid (FA) fortification, found no excess NTDs or other major malformations in more than 4600 periconception DTG exposures (Gill et al., 2023). A US study of medical claims covering 14 million pregnancies detected no additional NTD risk in more than 1000 periconception DTG exposures within a population protected by long-standing FA fortification and widespread prenatal supplementation (Kourtis et al., 2023). The same analysis, however, reported a significantly higher rate of pregnancy loss in women who conceived while receiving DTG and, to a lesser extent, other antiretrovirals, suggesting that early embryonic viability remains a potential vulnerability even in the absence of overt malformations. More recently, the same Eswatini network reported that adverse birth outcomes, including pregnancy loss, were elevated in women infected with human immunodeficiency virus (HIV) compared with HIV-negative women, yet were not linked to DTG or other antiretroviral classes (Gill et al., 2025). Loss of pregnancy is consistent with overt developmental toxicity observed in animal models exposed to DTG during early development. Together, these data suggest that maternal HIV infection and the underlying nutritional milieu, rather than DTG alone, drive much of the residual risk. Even so, the biological capacity of DTG to perturb embryogenesis remains unclear, and a mechanistic framework is essential to understand and mitigate risks. Rare teratogenic effects can elude even large epidemiologic datasets unless high-risk subgroups are prospectively identified; folate and magnesium (Mg) status vary widely across regions and could modulate hazard; and all current and next-generation integrase-strand-transfer inhibitors (INSTIs) share the same metal-chelating pharmacophore, so insights gained here will generalize across the class. Regulatory labeling and clinical counseling should rest on a clear understanding of drug–nutrient interactions.

Two non-exclusive hypotheses dominate current discussion. First, DTG has been shown to act as a partial antagonist of folate receptor 1 (FOLR1) *in vitro* and to trigger folate-responsive malformations in zebrafish embryos (Cabrera et al., 2019). Second, the antiviral activity of DTG depends on chelating the pair of Mg ions in the HIV-1 integrase active site (Cook et al., 2020). Analogous Mg sequestration in the embryo could either lower the pool of free Mg or alter FOLR1 activity, thereby compounding folate stress.

Mg deficiency is closely linked to embryonic malformations in multiple species. In humans, insufficient Mg levels are associated with an increased risk of NTDs (Groenen et al., 2004; Shaw et al., 1999). Mice lacking the Mg transporter Trpm6 exhibit NTDs due to impaired Mg transport by extraembryonic tissues (Chubanov et al., 2016; Walder et al., 2009). Similarly, *Xenopus* embryos deficient in either Trpm6 or Trpm7, both critical Mg transporters, fail to achieve proper neural tube closure (Komiya et al., 2017; Liu et al., 2011). The loss of cyclin M2 (Cnnm2), a protein that enhances Mg conductance through Trpm7, leads to hypomagnesemia and exencephaly in mice, and abnormal brain development in zebrafish (Arjona et al., 2014; Franken et al., 2021). Importantly, mouse pregnancies with low baseline Mg or subjected to low-Mg diets demonstrate heightened susceptibility to DTG-induced neural tube defects, whereas pregnancies with higher maternal Mg levels are protected (Gelineau-van Waes et al., 2023).

Dissecting the interplay between folate transport and Mg homeostasis is not only clinically relevant, it also promises fresh insight into fundamental requirements for early vertebrate development, for which maternal folate loading, transmembrane folate carriers and dynamic Mg gradients remain incompletely understood.

Important knowledge gaps remain: the extent to which Mg availability modulates DTG-induced teratogenicity *in vivo*, the mechanistic influence of Mg on DTG–FOLR1 interactions, and the mechanisms by which folate-signaling deficits influence embryonic susceptibility to DTG have yet to be defined.

Zebrafish provide an ideal system to address these gaps. Their externally developing embryos permit precise control of exposure windows, rapid high-content phenotyping and straightforward genetic manipulation. Importantly, DTG induces malformations in zebrafish only when exposure occurs during the first 4 h post-fertilization (hpf) (Cabrera et al., 2019), a developmental window that parallels the periconceptional period implicated in human epidemiology (Zash et al., 2018), supporting translational relevance. Here, we used this model to (1) quantify DTG toxicity and rescue by folate versus divalent cations in wild-type embryos, (2) test whether Mg modulates DTG–FOLR1 binding *in vitro*, and (3) determine DTG sensitivity and Mg rescue potential in maternal-zygotic *folr1* mutants that enter development with folate deficiency.

We show that DTG teratogenicity in zebrafish is mitigated by folate or Mg supplementation; that Mg abolishes the antagonism of DTG on purified FOLR1 *in vitro*; and that *folr1* mutants are hypersensitive to DTG yet cannot be rescued by exogenous folate but can be rescued by Mg, revealing a dual mechanism of FOLR1 antagonism and Mg sequestration. These findings identify Mg status as a modifiable determinant of DTG developmental risk, provide a mechanistic framework for the context-specific human data, and establish a rapid zebrafish platform for screening integrase inhibitors and probing fundamental links between Mg, folate transport and early vertebrate development.

## RESULTS

### DTG causes embryonic defects in zebrafish that are rescued by folate and Mg

**Fig. 1.**
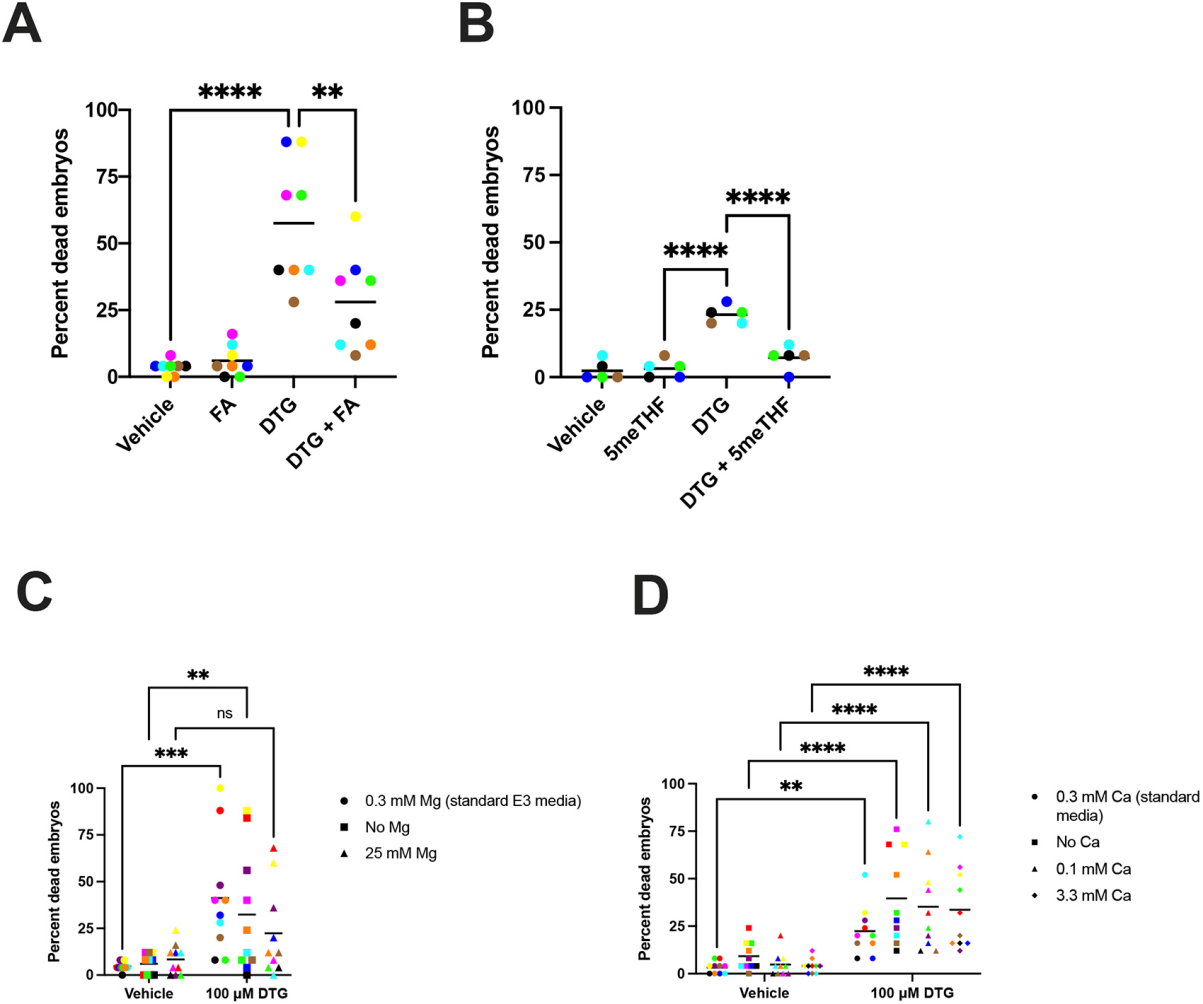
**Dolutegravir toxicity is rescued by folates and magnesium but not calcium.** (A,B) Wild-type zebrafish embryos exposed to 100 μM dolutegravir freebase (DTG) beginning at 2-4 h post-fertilization (hpf) displayed increased mortality at 1 day post-fertilization (dpf). Co-treatment with folic acid (FA; 600 ng/ml; A) or 5-methyltetrahydrofolate (5meTHF; 6 μg/ml; B) significantly reduced DTG-induced defects. A, *N*=8 biological replicates (clutches), *n*=800 embryos total (200 embryos per exposure); B, *N*=5 replicates, *n*=500 embryos total. One-way ANOVA with Tukey's multiple comparisons test. (C) Magnesium (Mg) supplementation (25 mM) rescued developmental toxicity of DTG-exposed embryos. *N*=10 replicates, *n*=1500 embryos total. (D) Calcium (Ca) had no protective effect. *N*=10 replicates, *n*=2000 embryos total. C,D, two-way ANOVA with Tukey's multiple comparisons test. For all graphs, each data point represents the mean percentage of normal embryos from a single clutch of 25 embryos (each clutch of embryos was produced from different parents). Within each graph, data points of the same color are from the same clutch. **P<0.002; ***P<0.0002; ****P<0.0001; ns, not significant.

We exposed wild-type zebrafish embryos to 100 µM DTG or vehicle beginning at 2-4 hpf and assayed whether embryos were normal, dead or exhibited morphologic abnormalities at 24 hpf. Compared to vehicle controls, embryos exposed to DTG displayed increased mortality at 1 day post-fertilization (dpf) (Fig. 1A), consistent with previous results (Cabrera et al., 2019). Co-treatment with 600 ng/ml FA or 6 μg/ml 5-methyltetrahydrofolate (5-meTHF) significantly reduced DTG-induced mortality [FA: vehicle, 4±3% (mean±s.d.) dead embryos; vehicle+FA, 6±6%; DTG, 58±24%; DTG+FA, 28±18%; *N*=8, *n*=800 embryos total; 5meTHF: vehicle control, 2±4% dead embryos; vehicle+5meTHF, 3±3%; DTG, 23±3%; DTG+5meTHF, 7±4%; *N*=5 clutches, *n*=500 embryos total]. DTG exposure caused a concomitant reduction in the percentage of normal embryos, which was rescued by FA and 5meTHF (Fig. S1). There were few or no abnormal embryos present (0-3% in DTG exposure group, 0% in all other groups); embryos were either alive and grossly normal, or dead (Fig. S1A,B). Concentrations of FA were selected based on previous results (Cabrera et al., 2019). Concentration of 5-meTHF was selected to be 10× that of FA based on previous studies of its endogenous levels (Kao et al., 2013) and the fact that it was nontoxic (Fig. 1B).

Mg (25 mM) supplementation partially rescued the mortality of DTG-exposed embryos. We observed a statistically significant increase in the percentage of dead embryos in DTG versus vehicle in the presence of embryo media containing no Mg or 0.3 mM Mg (the standard concentration in embryo media), while there was no statistically significant increase in the mean percentage of dead embryos between DTG and vehicle in the presence of 25 mM Mg (Fig. 1C; vehicle control in 0.3 mM Mg, 5±3% dead embryos; DTG in 0.3 mM Mg, 41±31%; vehicle control 25 mM Mg, 8±8%; DTG in 25 mM Mg, 22±24%; *N*=10 clutches, *n*=1500 embryos total). The reduction in dead embryos in the presence of 25 mM Mg occurred alongside a statistically significant reduction in the percentage of abnormal embryos and an increase in the percentage of normal embryos (Fig. S1C; vehicle control 0.3 mM Mg, 0±0% abnormal embryos; DTG in 0.3 mM Mg, 17±13%; vehicle control in 0 mM Mg, 0±0%; DTG in 0 mM Mg, 21±13%; vehicle control 25 mM Mg, 8±8%; DTG in 25 mM Mg, 6±6%; vehicle control 0.3 mM Mg, 95±3% normal embryos; DTG in 0.3 mM Mg, 41±20%; vehicle control 0 mM Mg, 94±5%; DTG in 0 mM Mg, 47±25%; vehicle control 25 mM Mg, 92±8%; DTG in media with 25 mM Mg, 72±21%).

In contrast to Mg, calcium (Ca) had no protective effect on mortality (Fig. 1D; vehicle control in 0.3 mM Ca, 3±3% dead embryos; DTG in 0.3 mM Ca, 22±13%; vehicle control 3.3 mM Ca, 4±4%; DTG in 3.3 mM Ca, 34±21%; *N*=10 clutches, *n*=2000 embryos total). Ca levels also had no statistically significant effect on percentages of normal or abnormal embryos (Fig. S1D; vehicle control in 0.3 mM Ca, 97±3% normal embryos; DTG in 0.3 mM Ca, 56±21%; vehicle control 3.3 mM Ca, 96±4%; DTG in 3.3 mM Ca, 46±23%; vehicle control in 0.3 mM Ca, 0±0% abnormal embryos, DTG in 0.3 mM Ca, 4±7%, vehicle control in 3.3 mM Ca, 0±0%, DTG in 3.3 mM Ca, 7±7%). These results indicate that the teratogenic effects of DTG are folate responsive and can be mitigated by increasing available folate or increasing Mg concentrations, consistent with the hypothesis that the partial antagonism of FOLR1 by DTG is mitigated by Mg.

We observed variability in the penetrance of DTG-induced phenotypes across clutches. To reduce variability attributable to baseline clutch quality, analyses were restricted to clutches in which ≥90% of embryos were phenotypically normal following vehicle exposure.

We evaluated potential contributors to variability (Table S1). Parental age was not associated with the proportion of normal (*R*²=0.007, *P*=0.62), dead (*R*²=0.060, *P*=0.15) or abnormal embryos (*R*²=0.064, *P*=0.14). Regression analysis of outcome measures versus date of experiment (*n*=36 clutches) revealed no association with the percentage of normal embryos (*R*²=0.012, *P*=0.52). However, the percentage of abnormal embryos increased over time (*R*²=0.49, *P*=2.3×10^−^⁶), whereas the percentage of dead embryos decreased over time (*R*²=0.31, *P*=0.00047). These findings indicated a temporal shift in the distribution of abnormal versus dead outcomes, without a change in the proportion of embryos classified as normal. Thus, although the severity profile of affected embryos varied over time, the overall fraction of embryos remaining phenotypically normal following DTG exposure remained stable. All treatment comparisons were performed within experiments using embryos derived from the same clutch and assessed concurrently.

### Mg reduces DTG-mediated inhibition of FOLR1 *in vitro*

Previous work from our laboratory showed that DTG inhibits FOLR1 through a non-competitive mechanism, meaning that DTG does not directly compete with folate for the same binding site but instead reduces folate binding by altering receptor function (Cabrera et al., 2019). A separate study showed that Mg supplementation (e.g. high Mg diet) reduced the incidence of DTG-associated neural tube defects in mice, suggesting that Mg modifies DTG–FOLR1 interactions (Gelineau-van Waes et al., 2023). To test this directly, we examined folate binding to purified bovine FOLR1 (bFOLR1) *in vitro* in the presence of DTG and increasing concentrations of Mg.

We performed competitive folate binding assays using a fixed concentration of horseradish peroxidase-conjugated folic acid (FA-HRP) and increasing concentrations of unlabeled folic acid (FA). After removal of unbound ligand, FA-HRP bound to immobilized bFOLR1 was quantified by chemiluminescence. In this assay, signal intensity reflected the amount of FA-HRP bound to bFOLR1. Signal intensity decreased as increasing amounts of unlabeled FA competed for receptor binding. From these data, we calculated the half-maximal inhibitory concentration (IC50) of unlabeled FA, defined as the concentration of FA required to reduce FA-HRP binding by 50%.

**Fig. 2.**
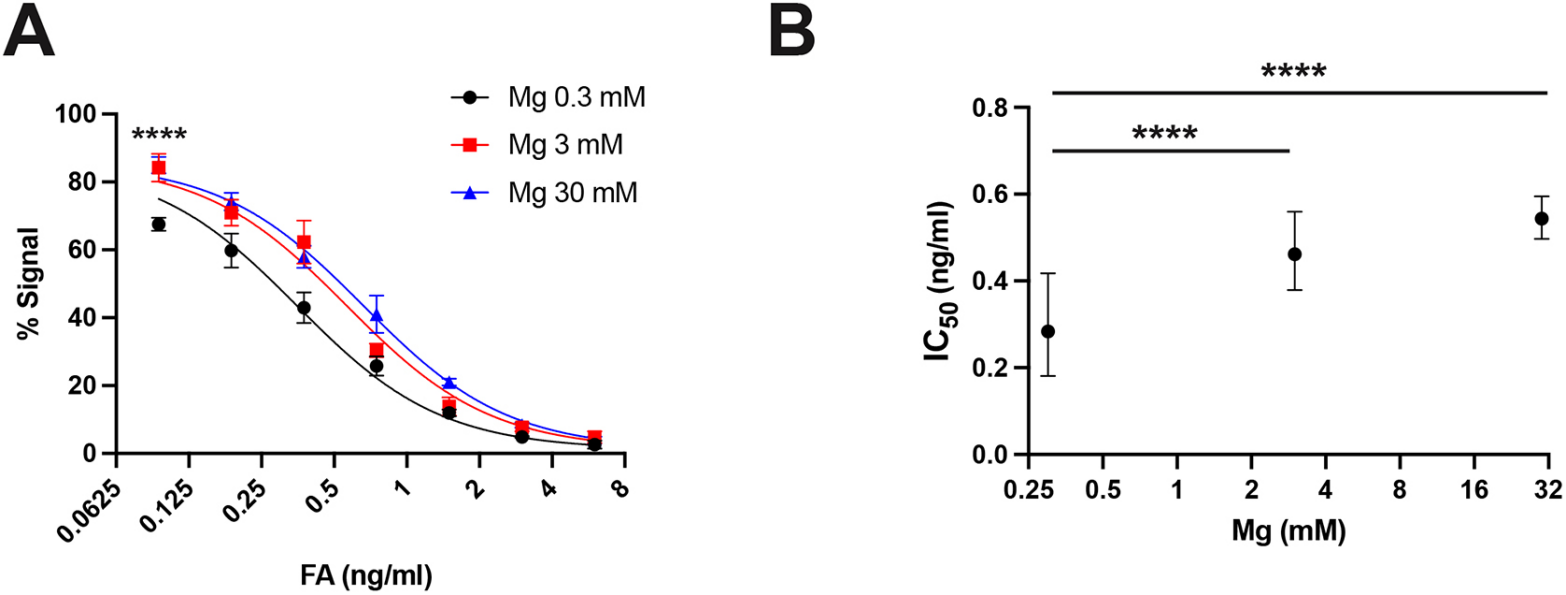
**Magnesium increases folic acid binding to folate receptor in the presence of DTG.** (A) *In vitro* measurements of folate binding to purified bovine FOLR1 (bFOLR1) protein in the presence of 100 μM DTG together with 0.3-30 mM Mg. Folate binding is shown as normalized signal intensity (*y*-axis). Increasing concentrations of unlabeled folic acid (FA; *x*-axis) reduces signal as it competes with labeled folic acid (HRP-FA). Curves fit using least squares regression, *R*^2^=0.99 (30 mM Mg), 0.96 (3 mM), 0.95 (0.3 mM), *n*=4, mean±s.d.. At maximum signal (0.09 ng/ml FA), there is a statistically significant difference in the signal with 0.3 mM Mg versus 3 or 30 mM Mg. One-way ANOVA, Dunnett's multiple comparisons test, ****P<0.0001. (B) Data from A were used to calculate the half-maximal inhibitory concentration (IC50) of unlabeled FA blocking binding of HRP-FA to bFOLR1. Increased Mg reduced the IC50. Error bars=95% confidence interval. Sum of squares F test, ****P<0.0001. *X*-axes are scaled to log2.

To determine whether Mg alters DTG-mediated inhibition of folate binding, assays were performed in the presence of 100 µM DTG and low (0.3 mM), intermediate (3 mM) or high (30 mM) Mg. At a fixed concentration of unlabeled FA for which the binding signal is highest (0.09 ng/ml FA), FA-HRP binding to bFOLR1 was significantly higher in the presence of 3 mM or 30 mM Mg than with 0.3 mM Mg (Fig. 2A). Thus, increasing Mg concentrations partially restored folate binding to bFOLR1 despite the presence of DTG.

Consistent with this observation, increasing Mg concentrations resulted in a progressive increase in the IC50 for unlabeled FA, meaning that it takes more FA to block FA-HRP binding to FOLR1 (in the presence of 100 µM DTG) when Mg concentrations increase (Fig. 2B). Importantly, this rightward shift in IC50 does not indicate competitive antagonism between Mg and folate (i.e. Mg and folate do not compete for binding to bFOLR1) but instead reflects increased folate–receptor binding capacity in the presence of Mg despite continued DTG exposure. When Mg is present, higher concentrations of unlabeled FA are required to displace FA-HRP from bFOLR1. In other words, Mg reduces the ability of DTG to inhibit folate binding, thereby strengthening FA–bFOLR1 interactions even though DTG remains present. Together, these data indicate that Mg functionally reverses DTG-mediated inhibition of folate receptor binding *in vitro*. The results are consistent with a model in which Mg modifies the allosteric inhibition of FOLR1 by DTG, restoring folate binding rather than directly competing with folate for the receptor. We conclude that Mg increases folate binding to bFOLR1 in the presence of DTG, supporting the hypothesis that Mg blocks the antagonistic effects of DTG at FOLR1.

### Generation and characterization of folate receptor mutant embryos

FOLR1 is present on the extracellular membrane, where it binds folates such as FA and 5meTHF and brings them into the cell via receptor-mediated endocytosis (Lacey et al., 1989; Elwood, 1989; Piedrahita et al., 1999). If DTG acts as a FOLR1 antagonist, then we would expect *folr1* mutants to be resistant to DTG toxicity, although the mutants might have other phenotypes due to low endogenous folate levels. To directly test the role of the folate receptor in modulating DTG toxicity, we used CRISPR-Cas9 to generate zebrafish with mutations in the folate receptor gene *folr1*. We generated homozygous zebrafish with a mutation that introduces a premature termination codon after amino acid 131 (*bcm44* allele). Zygotic homozygous *folr1^bcm44^* embryos were grossly normal, viable to adulthood and fertile. Lack of a phenotype in zygotic embryos could be due to the presence of maternally deposited folates, which provide adequate folate levels during development.

**Fig. 3.**
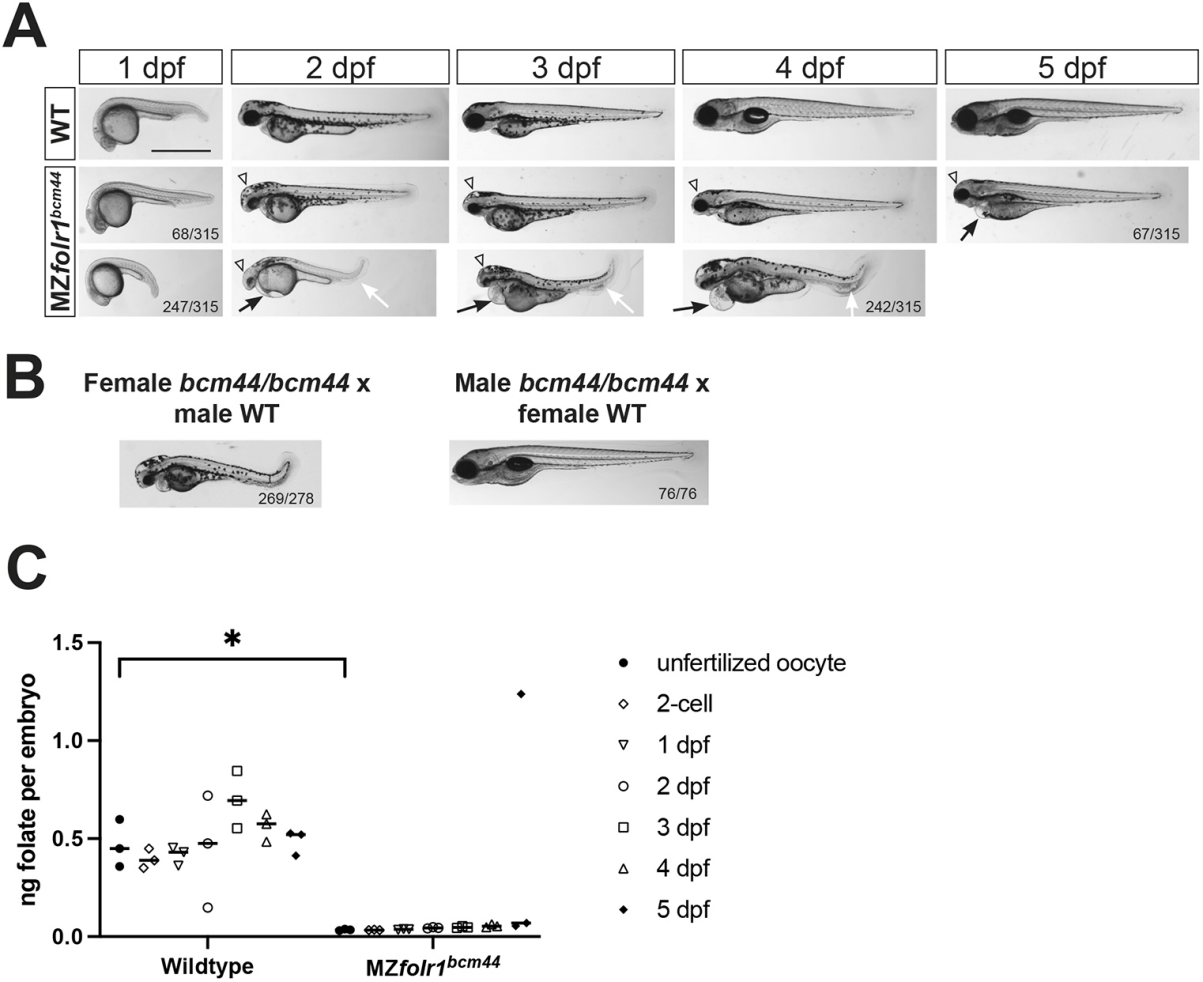
**Folate receptor mutant embryos have developmental abnormalities and reduced endogenous folate levels.** (A) Maternal zygotic homozygous (MZ*folr1*) mutants, without any drug exposure, display phenotypes such as smaller head (arrowheads), cardiac edema (black arrows) and curved tails with ventral thickening (white arrows) through 5 dpf. Examples of mutant phenotypes of varying severity are shown (middle and bottom rows). Scale bar: 1 mm (applies to A and B). WT, wild-type embryos. (B) *folr1* mutant phenotypes are maternally derived. Female homozygotes crossed to wild-type males produce embryos with phenotypes, whereas wild-type females crossed to homozygous males produce normal embryos. Lateral views with anterior to the left, dorsal to the top. Fractions in the bottom right corners refer to the number of embryos with the indicated phenotype over the total number of embryos examined from four clutches (female *bcm44×*WT) or from two clutches (male *bcm44×*WT). (C) We measured endogenous folate content in MZ*folr1^bcm44^* oocytes and embryos from fertilization through 5 dpf to assess how loss of the folate receptor affects folate levels. MZ*folr1* mutants possess significantly lower total folate levels in oocytes and embryos than do wild-type siblings, consistent with impaired maternal folate uptake during oogenesis and early development. Each dot is folate levels from a pool of 50 embryos. Two-way ANOVA with Tukey's multiple comparisons test, *P<0.05.

To generate maternal zygotic homozygous (MZ*folr1*) mutants, we crossed homozygous *folr1^bcm44^* adults to each other. At 1 dpf, 78% of MZ*folr1* mutants exhibited developmental delay but were otherwise grossly normal (247 out of 315 embryos); 22% were normal with no developmental delay (68 out of 315 embryos from four different clutches; Fig. 3A). Of the 247 MZ*folr1* mutants that exhibited developmental delay at 1 dpf, all went on to develop small head, cardiac edema, and curved tails with ventral thickening through 4 dpf (Fig. 3A). Five embryos died by 4 dpf, and the rest died by 5 dpf. Of the 68 MZ*folr1* mutants that appeared grossly normal (no delay) at 1 dpf, all went on to develop small heads and cardiac edema through 5 dpf (one embryo died; Fig. 3A). These phenotypic abnormalities mirror aspects of folate deficiency in mammalian embryogenesis and suggest that the folate receptor is essential for accumulating sufficient folates for normal development in zebrafish. To confirm that these *folr1* phenotypes are maternally derived, we crossed adult homozygous *folr^bcm44^* females to wild-type males, and wild-type females to homozygous *folr^bcm44^* males, and assayed morphologic abnormalities in embryos through 5 dpf. We found that embryos derived from homozygous mothers exhibited phenotypes similar to those of MZ*folr^bcm44^* embryos, whereas embryos derived from homozygous fathers appeared normal (Fig. 3B). This result supports the idea that female zebrafish deposit folates into oocytes, which are critical for normal embryonic development.

MZ*folr1* embryos should also exhibit low folate levels compared to wild-type embryos. We measured endogenous folate content in MZ*folr1^bcm44^* oocytes and in MZ*folr1^bcm44^* embryos from fertilization through 5 dpf to assess how loss of the folate receptor affects folate status. Folate quantification revealed that MZ*folr1* mutants possess significantly lower total folate levels in oocytes and embryos than do wild-type embryos, consistent with impaired maternal folate uptake during oogenesis and early development (Fig. 3C). Together, our results provide a genetic validation that, in zebrafish, folate receptor function (and by extension folate transport) is crucial for normal development.

### Folate receptor mutant embryos exhibit heightened sensitivity to DTG and are not rescued by folate

**Fig. 4.**
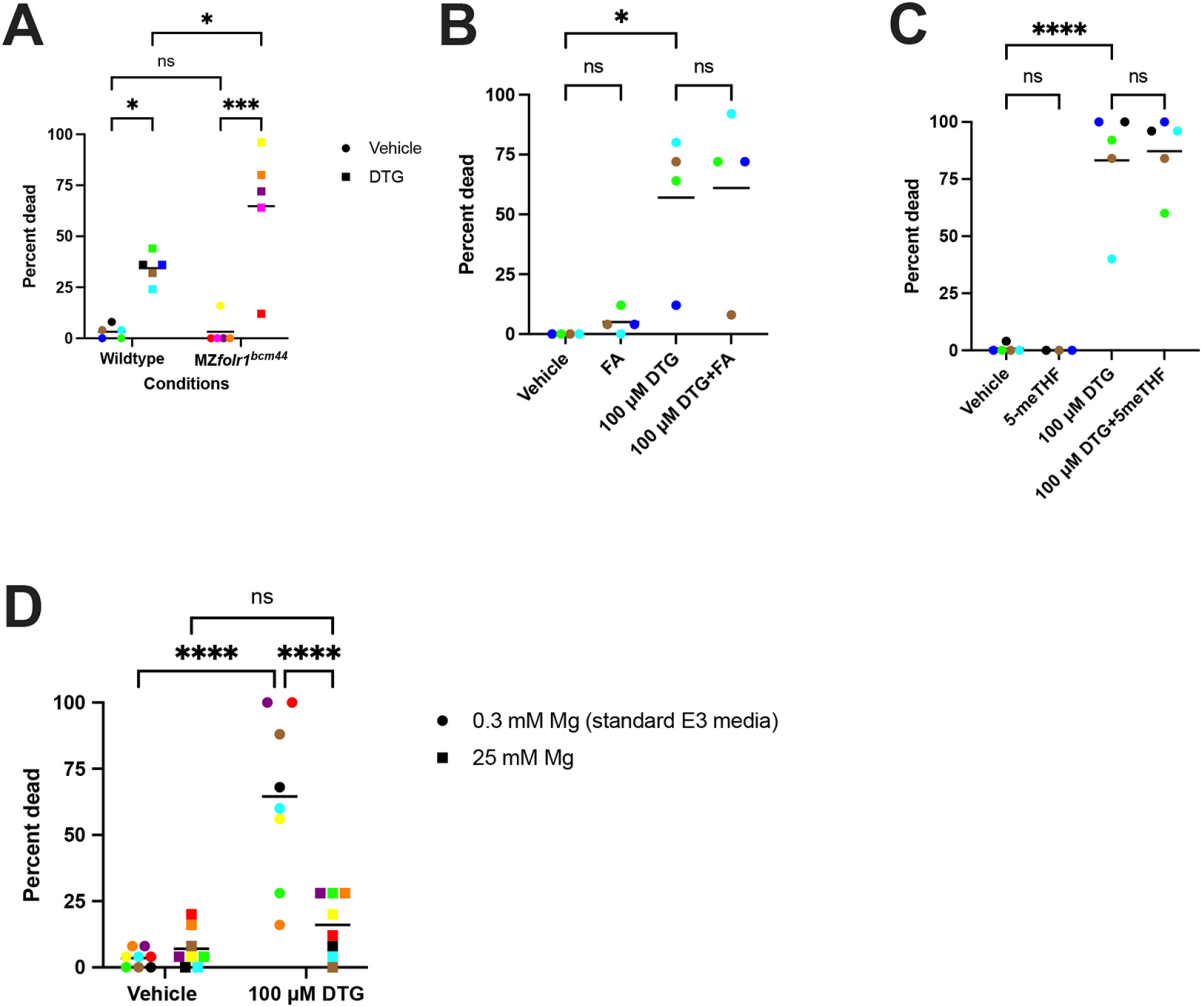
**Folate receptor mutants exhibit heightened sensitivity to DTG and are not rescued by folates.** To directly test the role of the folate receptor in modulating DTG toxicity, we utilized zebrafish carrying a maternal-zygotic mutation in *folr1* predicted to cause loss of functional protein (MZ*folr1^bcm44^*). (A) Following exposure to 100 μM DTG beginning at 2-4 hpf, MZ*folr1* mutants showed significantly greater death than did wild-type embryos at 1 dpf. Two-way ANOVA with Tukey's multiple comparisons test. *N*=5 biological replicates (clutches), *n*=500 embryos total. (B,C) Co-treatment with FA (600 ng/ml; B) or 5meTHF (6 μg/ml; C) failed to rescue DTG toxicity in MZ*folr1* mutants. B, *N*=4 replicates, *n*=400 embryos total; C, *N*=5 replicates (except 5-meTHF, *N*=3 replicates), *n*=425 embryos total. One-way ANOVA with Tukey's multiple comparisons test. (D) High Mg partially rescues DTG toxicity in MZ*folr1* embryos. *N*=8 replicates, *n*=800 embryos total. Two-way ANOVA with Tukey's multiple comparisons test. For every graph, each data point represents the mean percentage of dead embryos from a single clutch of 25 embryos. Within each graph, data points of the same color are from the same clutch. *P<0.05, ***P<0.0002; ****P<0.0001; ns, not significant.

To directly test the role of the folate receptor in modulating DTG toxicity, we exposed MZ*folr1^bcm44^* mutant embryos to DTG beginning at 2-4 hpf and assayed developmental phenotypes at 1 dpf. Because untreated MZ*folr1^bcm44^* embryos are either grossly normal or exhibit developmental delay (but are otherwise grossly normal) at 1 dpf, we assayed whether DTG exposure would cause more severe morphologic phenotypes or death at 1 dpf. Following exposure to DTG (100 µM), MZ*folr1* mutants showed significantly greater death at 24 hpf than did wild-type embryos at equivalent DTG concentrations (Fig. 4A; wild-type vehicle, 3±3% dead embryos; MZ*folr1^bcm44^* vehicle, 3±7%; wild-type DTG, 34±7%; MZ*folr1^bcm44^* DTG, 65±32%; *N*=5 clutches, *n*=500 embryos total). Whereas DTG-induced developmental toxicity in wild-type embryos could be rescued by co-treatment with FA (600 ng/ml) or 5-meTHF (6 µg/ml), folate supplementation had minimal to no protective effect in MZ*folr1* mutants exposed to DTG (Fig. 4B, MZ*folr1^bcm44^* FA experiments: vehicle, 0±0% dead embryos; FA, 5±5%; DTG, 57±31%; DTG+FA, 61±37%; *N*=4 clutches, *n*=400 embryos total; Fig. 4C, 5-meTHF experiments: vehicle, 1±2% dead embryos; 5-meTHF, 0±0%; DTG, 83±25%; DTG+5-meTHF, 87±16%; *N*=3-5 clutches, *n*=425 embryos total). These findings indicate that a functional folate receptor is required for the rescue effect of exogenous folate and that loss of FOLR1 exacerbates the developmental toxicity of DTG. The inability to rescue folate receptor mutants with supplemental folate further supports that the teratogenic effects of DTG occurs via blocking folate–FOLR1 interactions (rather than downstream folate metabolism). The folate-deficient state of MZ*folr1* embryos, noted above, likely predisposes them to additional stressors, explaining their increased susceptibility to DTG at 1 dpf.

### Mg partially rescues DTG toxicity in folate receptor mutant embryos

Given that exogenous folate cannot rescue *folr1* mutants, we tested whether Mg modifies DTG toxicity in *folr1* mutants. High Mg (25 mM) partially rescued DTG-exposed MZ*folr1* embryos (Fig. 4D; vehicle, 4±3% dead embryos; 100 µM DTG, 65±31%; vehicle+Mg, 7±7%; 100 µM DTG+Mg, 16±11%; *N*=8 clutches, *n*=800 embryos total). The improvement from DTG in 0.3 mM Mg (standard media) to DTG in 25 mM Mg was statistically significant (*P*<0.002, two-way ANOVA), indicating that Mg mitigates DTG developmental toxicity in the absence of Folr1 function. These data place the free (unchelated) DTG versus Mg-bound DTG balance upstream of folate transport and reveal a Folr1-independent component of DTG embryotoxicity.

## DISCUSSION

### DTG chelates Mg and impairs folate receptor function, leading to folate insufficiency and developmental toxicity

**Fig. 5.**
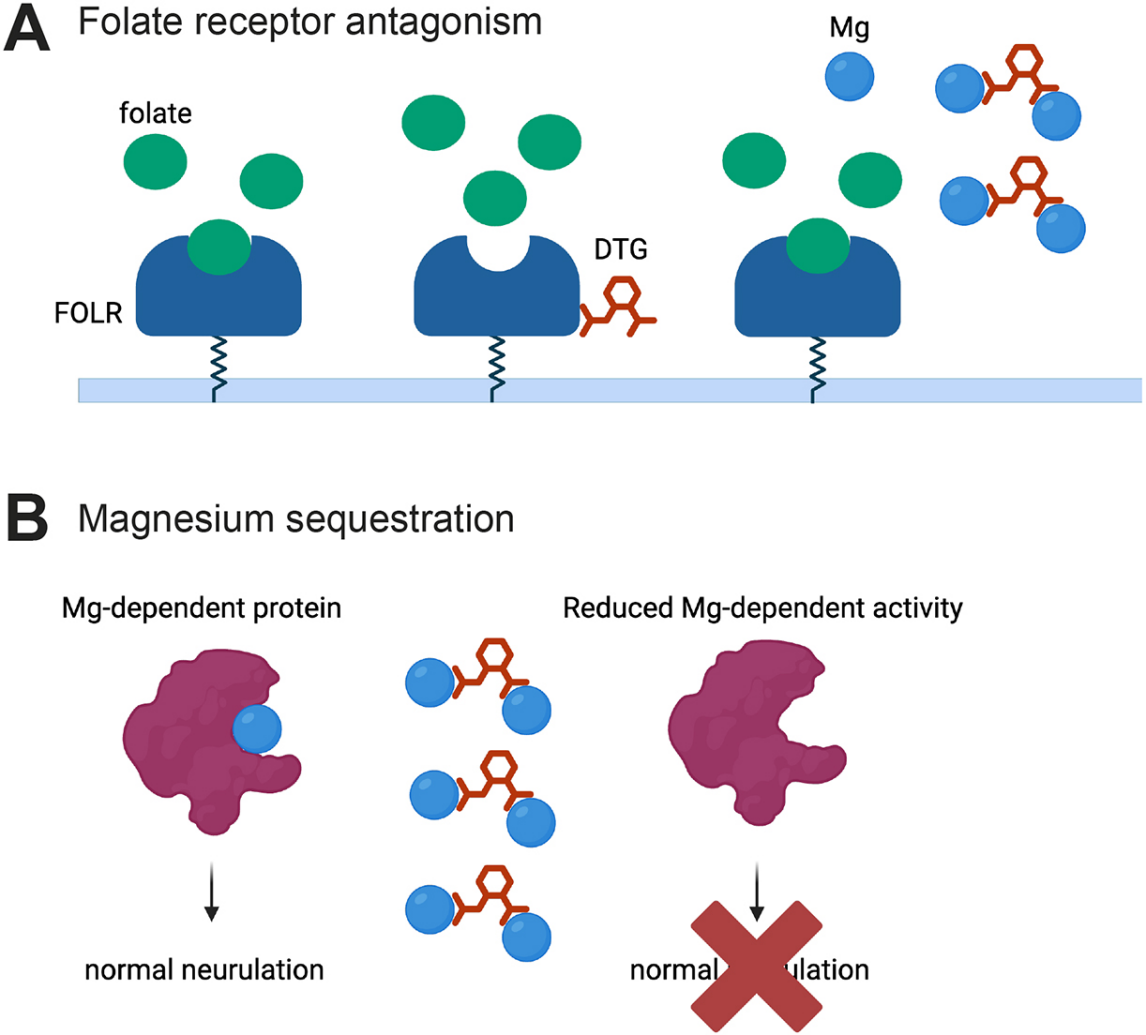
**Conceptual model illustrating two mechanisms by which DTG may perturb neurulation and embryonic development.** (A) DTG binds folate receptor (FOLR) at a site distinct from the folate binding site, reducing folate–FOLR interactions. Increasing Mg shifts DTG toward an Mg-bound complex, decreasing the pool of free DTG available to engage FOLR and restoring folate binding. (B) DTG may also sequester Mg through chelation, reducing Mg availability for Mg-dependent proteins required for normal neurulation. This schematic illustrates a model consistent with the *in vitro* binding data and the Mg-dependent rescue observed *in vivo*. Created in BioRender by Gorelick, D. A. (2026). https://BioRender.com/ynl7loz. This figure was sublicensed under CC-BY 4.0 terms.

Integrating our findings, we propose a mechanistic model in which the teratogenic effects of DTG stem from its Mg-chelating activity coupled with partial antagonism of the folate receptor. DTG can bind to Mg ions, which not only is the basis for its antiviral action – chelating Mg ions in the active site of HIV-1 integrase, thus blocking the reaction that would splice viral DNA into the host genome (DeAnda et al., 2013; Hare et al., 2011; Passos et al., 2020) – but also for chelating and sequestering Mg required for embryonic development (Gelineau-van Waes et al., 2023; Song et al., 2015). By chelating Mg, DTG alters folate receptor interactions as indicated by altered folate binding, effectively reducing folate uptake into the developing embryo. This results in a functional folate deficiency during critical stages of development, causing embryonic phenotypes. Supplemental folate (FA or 5-meTHF) can overcome this blockade in wild-type embryos by mass action, flooding the system with folate to utilize any remaining uptake capacity. Likewise, extra Mg can saturate the chelation capacity of DTG, preserving folate receptor function and normal Mg-dependent cellular processes, thereby rescuing the embryo from the toxic effects of DTG. In *folr1* mutants, exogenous folate cannot rescue because transport via Folr1 is reduced or absent. However, high Mg partially rescued DTG toxicity in *folr1* mutants. This indicates a Folr1-independent component of DTG toxicity and supports a model in which the balance between free DTG and Mg-bound DTG acts upstream of folate transport. Mg binds free DTG, producing a chelated form with limited solubility, bioavailability and affinity for FOLR1 and simultaneously supports Mg-dependent (Mg>DTG) cellular processes. Fig. 5 illustrates a conceptual model integrating these findings, highlighting two proposed mechanisms by which DTG may perturb embryonic development: antagonism of folate receptor function and sequestration of Mg required for Mg-dependent proteins.

### Mechanistic considerations of Mg–DTG interactions

Although our data support a model in which Mg modifies DTG-associated folate pathway disruption, the precise molecular mechanism by which Mg exerts this protective effect remains unresolved. The *in vitro* binding assays used here directly measure FA–FOLR1 interactions in the presence of DTG and Mg but do not directly assess DTG–FOLR1 binding or Mg–DTG complex formation. As a result, the data cannot distinguish whether Mg primarily acts by altering DTG availability or efficacy (for example, through chelation that reduces free DTG), by modifying FOLR1 conformation or susceptibility to DTG-mediated antagonism, or through a combination of these effects.

Importantly, our results argue against a model in which Mg directly competes with folate for FOLR1 binding. Instead, Mg restores folate binding in the presence of DTG, consistent with a reduction in the functional impact of DTG on folate receptor activity. The partial rescue of DTG toxicity in *folr1* mutant embryos further supports the existence of a Folr1-independent component of Mg protection, placing Mg action upstream of, or parallel to, folate transport itself. Together, these findings indicate that the balance between free DTG and Mg-bound DTG, rather than folate competition, is a key determinant of developmental outcome.

### Why Mg, but not Ca, protects embryos from DTG toxicity

Our findings that Mg, but not Ca, protects zebrafish embryos from DTG toxicity are supported by biochemical and structural studies of DTG, which show that DTG binds Mg more effectively than Ca. Previous work from our laboratory demonstrated in biochemical and cellular assays that divalent cations can influence DTG–FOLR1 interactions and that Ca can modulate folate binding to FOLR1 (Cabrera et al., 2019). These findings illustrate that Ca can affect folate–receptor interactions under *in vitro* conditions. However, our *in vivo* embryo experiments show that supplementing Mg, but not Ca, protects against DTG-induced developmental toxicity, suggesting that Mg coordination with DTG is the dominant mechanism operating in the embryo. We lack structures of DTG bound to FOLR1; however, we can infer DTG preference for Mg over Ca from structures of DTG bound to HIV-1 integrase. DTG chelates two divalent cations, like the twin Mg ions in the integrase active site. High-resolution structures of DTG bound to integrase and viral DNA show DTG binding both catalytic Mg ions. Attempts to substitute Ca abolish DNA strand-transfer activity because the larger Ca ion cannot fit the pocket or achieve the geometry (Hare et al., 2010, 2011; Passos et al., 2020). In free solution, the preference is echoed. Pharmacokinetic studies showed that Mg forms a soluble Mg–DTG complex, whereas Ca tends to generate an insoluble precipitate that lowers oral DTG exposure but does not leave a stable, soluble Ca–DTG species in equilibrium (Song et al., 2015). DTG can interact with Ca, enough to reduce oral bioavailability, but the affinity is far lower than for Mg, and the resulting complex is poorly soluble. By contrast, DTG forms a tight, well-soluble chelate with Mg that is the same species captured in every crystallographic structure of DTG-bound integrase.

### Convergence with cation-chelation toxicology

Independent work on intrathecally or intracranially delivered oligonucleotides (ONs) shows that high local ON concentrations can chelate endogenous divalent cations, producing nonspecific phenotypes (side effects) in rodents and non-human primates (Bravo-Hernandez et al., 2026; Miller et al., 2024; Moazami et al., 2024). Increasing Mg in the dosing solution mitigated these acute nonspecific effects without impairing ON uptake or activity. These studies attributed the nonspecific toxicity to cation chelation and showed mitigation by raising the Mg:drug ratio. Although the modality and compartment differ from our zebrafish system (intrathecal or intracranial dosing of the central nervous system versus whole-embryo bath exposure), the shared principle is that providing Mg in the exposure medium or dosing formulation can pre-emptively convert DTG to DTG–Mg–chelate, reducing DTG–folate–receptor interactions, which aligns with our findings that Mg reduces the ability of DTG to antagonize FOLR1 *in vitro* and rescues embryos *in vivo*.

### Translational relevance and human pregnancy data

The requirement that DTG be present only during the first 4 hpf to elicit toxicity in zebrafish (Cabrera et al., 2019) mirrors the narrow periconceptional neurulation window implicated in the Botswana studies (Zash et al., 2018, 2019). A cohort in Eswatini found no excess structural defects among periconception DTG exposures (Gill et al., 2023, 2025), whereas a US claims study, conducted in a folate-fortified setting, also reported no additional NTD risk but did detect a significantly higher rate of pregnancy loss in women who conceived while on DTG and a smaller, yet similar, increase with other antiretrovirals (Kourtis et al., 2023). Pregnancy loss, stillbirth and low birthweight were likewise elevated in the Eswatini cohort regardless of antiretroviral regimen (Gill et al., 2025). Developmental toxicology classifies embryonic death as one of the four canonical manifestations of deviant development. The 24 hpf mortality we observe in DTG-exposed zebrafish thus provides an experimental analog of these early human losses, while surviving embryos display the malformations noted in the original Botswana signal. Partial rescue of *folr1* mutants by Mg underscores that maternal Mg status can buffer DTG-associated risk even when folate transport via Folr1 is compromised. By defining how Mg and folate modulate DTG toxicity, our work provides a mechanistic framework for interpreting these context-specific human data and for guiding targeted surveillance and supplementation strategies.

### Implications for pre-clinical screening

Our data indicate that early zebrafish embryos offer a tractable, 24-h read-out of DTG-induced malformations that is sensitive to both folate and Mg modulation. The combination of external development, optical transparency and small-molecule permeability enables high-throughput, low-cost testing of drug analogs in a whole-vertebrate context. With automated imaging and computer-vision scoring, the assay could be scaled to 96-well or 384-well format, allowing side-by-side comparison of current and next-generation INSTIs for teratogenic potential. Importantly, the platform captures chemical features that influence folate-receptor antagonism and those that affect Mg binding, providing a unique opportunity to rank order compounds by developmental safety margins. A zebrafish screen could serve as an early gate to identify integrase inhibitors with reduced developmental risk and to inform medicinal-chemistry optimization of the INSTI scaffold for preclinical testing.

### Limitations

First, the DTG concentration used in embryo media (100 µM) exceeds typical human plasma levels. Without direct pharmacokinetic measurements in zebrafish, we cannot determine the actual intra-embryonic DTG levels. It is possible that supraphysiologic DTG concentrations in embryo media are needed to achieve physiological concentrations in embryos, as has been shown to occur with other small molecules such as estrogens and androgens (Souder and Gorelick, 2017, 2018; Zadmajid et al., 2024). Second, our conclusion that Mg modifies DTG developmental toxicity is based on rescue experiments *in vivo*, including partial rescue in *folr1* mutants, and on FOLR1 binding data *in vitro*. We did not measure intracellular free or total Mg in embryos, so the Mg-dependent protection we propose (conversion of free DTG to Mg-bound DTG chelate and support of Mg-dependent processes by increasing the Mg:DTG ratio) remains inferential. Third, we assessed DTG antagonism only at Folr1 and did not quantify the effects of DTG on other zebrafish folate transporters (reduced folate carrier Slc19a1, proton-coupled folate transporter Slc46a1, mitochondrial folate transporter Slc25a32). Thus, the relative importance of each transporter in mediating or modifying DTG toxicity is unresolved.

## Conclusions

In summary, our model posits that the developmental toxicity of DTG is driven by a dual mechanism of (1) folate pathway inhibition (via FOLR1 antagonism) and (2) Mg sequestration, ultimately impairing essential folate-dependent developmental pathways and other Mg-dependent processes necessary for normal embryonic development. *folr1* mutants are already folate limited, so they have no metabolic cushion against Mg sequestration that impairs Mg-dependent processes, making them appear more sensitive to DTG exposure. Our findings support a dual-pathway model in which free DTG antagonizes FOLR1, while the ability of DTG to chelate Mg also perturbs Mg-dependent biology. Partial rescue of MZ*folr1* embryos by Mg demonstrates that Mg acts upstream and independently of Folr1, highlighting the balance between free DTG and Mg-bound DTG as a key driver of developmental outcome. Our results suggest folate and Mg status as critical modifiers of DTG teratogenic risk in zebrafish embryos, in agreement with recent findings in mice (Gelineau-van Waes et al., 2023; Tukeman et al., 2024), supporting the idea that ensuring adequate folate and Mg levels are potential strategies for mitigating DTG-associated birth defects, including in settings of compromised folate transport. Larger cohort studies in diverse populations, particularly in settings with varying folate and mineral intake, would help clarify human risk. Additionally, routine monitoring of plasma folate and systematic evaluation of nutritional interactions in patients on DTG, especially during pregnancy, would be expected to provide actionable clinical guidance and help prevent adverse outcomes.

## MATERIALS AND METHODS

### Zebrafish

Zebrafish were raised at 28.5°C on a 14 h light/10 h dark cycle in the Baylor College of Medicine (BCM) Zebrafish Research Facility in a recirculating water system (Tecniplast USA). Wild-type zebrafish were the AB strain (Westerfield, 2000), and mutant lines were generated using the AB strain. All procedures were approved by the BCM Institutional Animal Care and Use Committee.

### Embryo collection

Adult zebrafish were allowed to spawn naturally in pairs or in groups. Embryos were collected during 20 min intervals to ensure precise developmental timing within a group. Embryos were placed in 60 cm^2^ Petri dishes at a density of no more than 100 per dish in E3B media (60×E3B: 17.2 g NaCl, 0.76 g KCl, 2.9 g CaCl_2_-2H_2_O, 2.39 g MgSO_4_ dissolved in 1 l Milli-Q water; diluted to 1× in 9 l Milli-Q water plus 100 µl 0.02% Methylene Blue) and then stored in an incubator at 28.5°C with a 14 h light/10 h dark cycle.

### Chemical exposures

Fertilized embryos were placed in six-well plates (25 embryos per well) with 3 ml medium per well. Chemical exposure began at 2-4 hpf. Embryos were incubated at 28.5°C in the dark and were assessed for developmental malformations and lethality at 24 hpf by light microscopy. All chemicals were purchased as follows: dolutegravir freebase (DTG; AChemBlock, catalogue 10313, lot AC34478A, purity 98%, CAS registry number 1051375-16-6); folic acid (FA; Millipore-Sigma, catalogue F8758, purity, 97%, CAS 59-30-3), L-5-methyltetrahydrofolate calcium salt (5-meTHF; Lianyungang Jinkang Hexin Pharmaceutical Company, Magnafolate Pro, purity 99%, CAS 151533-22-1). Stocks were made in 100% DMSO at 1000× and diluted in E3B embryo medium to the final concentration (1×) at the time of treatment: 100 µM DTG, 600 ng/ml FA, 6 µg/ml 5-meTHF. All vehicle controls were 0.1% DMSO. To test the effect of Mg and Ca concentrations on DTG toxicity, E3B media were prepared as above but with varying Mg or Ca concentrations, based on previous reports demonstrating minimal gross toxicity following exposure to 0-25 mM Mg (Arjona et al., 2019) and 0-3.3 mM Ca (Adams et al., 2005). For all chemical exposure studies, analyses were restricted to clutches in which ≥90% of embryos were phenotypically normal following vehicle exposure.

In each zebrafish embryo exposure experiment, a single well of the six-well plate (i.e. one treatment condition) was considered the biological replicate (*N*). Twenty-five embryos (*n*) were placed per well, and the percentage of embryos exhibiting each phenotype was calculated per well. Experiments were repeated four to ten times using embryos derived from different parental pairs. All treatment groups within a given experiment were derived from the same clutch and assessed concurrently. Thus, in Fig. 1A, comparisons among vehicle, DTG, FA and DTG+FA were performed using *N*=8 biological replicates per condition (25 embryos per well per replicate; 200 embryos per condition; 800 embryos total across conditions).

### Generation of *folr1* mutant zebrafish

Chemically synthesized gRNAs for *folr1* and purified Cas9 protein were obtained from Synthego Corporation. We generated gRNA targeting the following sequence in *folr1* exon 4: GGCTGATGAGTCGTGGCGCC**GGG** (protospacer adjacent motif in bold). One-cell-stage embryos were injected using glass needles pulled on a Sutter Instruments Fleming/Brown Micropipette Puller (model P-97) and a regulated air-pressure micro-injector (Harvard Apparatus, PL1-90). Each embryo was injected with a 1 nl solution containing 1 µl of 10 µM gRNA, 1 µl of 20 µM Cas9 protein, 2 µl of 1.5 M KCl and 1 µl Phenol Red. The volume of the mixture was increased to 10 µl with 1× microinjection buffer [10 mM Tris-HCl, 0.1 mM EDTA (pH 8.0) in nuclease-free H_2_O] and incubated at 37°C for 5-7 min before injection as described previously (Burger et al., 2016). Mixtures were injected into the yolk of each embryo. Injected embryos were raised to adulthood and crossed to wild-type fish (AB strain) to generate F1 embryos. F1 adults were sequenced via tail fin biopsies to identify mutations predicted to cause loss of functional protein. The *bcm44* allele contains a 1 bp insertion (bold underlined, GAGTCGTGGC**C**GCCGGGAGCG) predicted to cause a missense mutation at amino acid 116 followed by a premature termination codon after amino acid 131.

### Genomic DNA isolation

Individual embryos or tail biopsies from individual adults were placed in 100 µl ELB [10 mM Tris (pH 8.3), 50 mM KCl, 0.3% Tween 20] with 1 µl proteinase K (800 U/ml, NEB) in 96-well plates, one sample per well. Samples were incubated at 55°C for 2 h (embryos) or 8 h (tail clips) to extract genomic DNA. To inactivate Proteinase K, plates were incubated at 98°C for 10 min and stored at −20°C.

### Genotyping

PCR and melting curve analysis was performed as described previously (Parant et al., 2009). PCR reactions contained 1 µl LC Green Plus Melting Dye (BioFire Diagnostics), 1 µl Ex Taq Buffer (Takara Bio) and 0.8 µl dNTP Mixture (Takara Bio) (2.5 mM each), 1 µl of each primer (5 µM), 0.05 µl Ex Taq (Takara Bio), 1 µl genomic DNA and deionized water up to 10 µl. PCR was performed in a Bio-Rad CFX96 thermal cycler, using black and white 96-well plates (Bio-Rad, HSP9665). The PCR reaction protocol was 98°C for 1 min, then 34 cycles of 98°C for 10 s, 60°C for 20 s and 72°C for 20 s, followed by 72°C for 1 min. After the final step, the plate was heated to 95°C for 20 s and then rapidly cooled to 4°C. Melting curves were generated with a Bio-Rad CFX96 Real-Time System over a 70-95°C range and analyzed with Bio-Rad CFX Manager 3.1 software. All mutations were confirmed by sequencing. PCR primers to generate amplicons for high-resolution melting curve analysis were as follows: 5′-ACTAATGTGTGTGTTCAGGCTG-3′, 5′-CATACCAGCTCTCACAGTCC-3′. PCR primers to generate amplicons for sequencing were as follows: 5′-CTGATTCCTTCAGCATGTGTGG-3′, 5′-﻿TGCATGCTGGGAACAAGACA-3′.

### Live imaging

Developmental malformations were assessed by visual inspection using a dissecting microscope, with masking of groups for scoring as normal, abnormal or dead. Embryos were imaged with a Nikon SMZ25 microscope equipped with a Hamamatsu ORCA-Flash4.0 digital CMOS camera. Images were equally adjusted for brightness and contrast in Adobe Photoshop Creative Cloud.

### Detecting folate levels in zebrafish

Folate receptor binding studies were performed as previously described (Cabrera et al., 2008; Tukeman et al., 2024). Briefly, bovine folate receptor (Sigma-Aldrich) at a concentration of 50 μg/ml was deposited onto microtiter plates (Corning, 3361) using a 2×2 grid printed in a 100 nl of 1× phosphate buffer saline via a robotic liquid handler (iDOT). Plates were sealed with adhesive tape and incubated overnight at 4°C in a cold room. The following day (∼16 h), plates were returned to room temperature, and the adhesive tape was removed to begin processing. Plates were washed 3× with 1× Tris-buffered saline+Tween 20 (1× TBST, pH 8.0) (Sigma-Aldrich) prior to sample application. Samples were prepared by adding 1% ascorbic acid to 1× TBST (∼pH 4), prepared as a 10× concentrate, and added to fish in water to 1×, sonicating 50 fish embryos per sample for 30 min, followed by manual pipetting. Samples were heat denatured for 5 min at 95°C, spun at 10 ***g*** for 7 min to pellet, and supernatant was collected for folate determination. Supernatant with neutralized with 5% NaOH, ∼2 µl, to ∼pH 7.5, then 1:10 FA-HRP (Vitros) was added to the neutralized supernatant. Samples were incubated for 30 min on washed plates, washed 3× after incubation and then imaged with ELISA substrate (SuperSignal ELISA Femto Substrate, Thermo Fisher Scientific). Plates were imaged on a Quansys imager, and data were extracted and analyzed using 4-plex grids for the 2×2 arrays.

### Measuring folate binding to FOLR1 *in vitro*

Folate binding assays were conducted in the presence of a fixed concentration of DTG (100 μM) and varying concentrations of MgCl_2_ (0.3, 3 or 30 mM). A working solution of FA-HRP (Vitros) was prepared by diluting the stock solution 1:10 into 1× TBST for all tested conditions. DTG and Mg were added directly to the FA-HRP binding buffer prior to plate incubation. Samples were incubated in microtiter plates for 30 min at room temperature, followed by three washes with 1× TBST to remove unbound ligand. Bound FA-HRP was detected using a chemiluminescent substrate, and signal intensity was quantified, as described above. Binding curves were generated by plotting normalized signal intensity as a function of serial diluted unlabeled FA concentrations (0.09-6 ng/ml), with *x*-axes scaled to log2. Curves were fit using least–squares regression. IC50 values for unlabeled FA inhibition of FA-HRP binding to bFOLR1 were calculated from these curves to quantify the effect of Mg on folate receptor binding in the presence of DTG (GraphPad). Data are presented as mean±s.d. for binding curves (*n*=4), with IC50 values reported with 95% confidence intervals.

### Statistical analysis

All statistical analyses were performed using GraphPad Prism and Microsoft Excel. For embryo exposure experiments, the biological replicate (*N*) was defined as a single well containing 25 embryos, and the percentage of embryos classified as normal, abnormal or dead was calculated per well. Each replicate represented embryos derived from a distinct clutch.

For experiments involving a single independent variable (e.g. chemical treatment in Fig. 1), comparisons among groups were performed using one-way ANOVA followed by appropriate post hoc tests as indicated in the figure legends. For experiments involving two independent variables (e.g. genotype and chemical exposure in Fig. 4), two-way ANOVA was used to assess main effects and interaction effects. When significant effects were detected, post hoc multiple comparison tests were performed as specified in the corresponding figure legends. Data are presented as mean±s.d. unless otherwise indicated. Statistical significance was defined as *P*<0.05.

To assess potential sources of variability across clutches, linear regression analyses were performed to evaluate associations between outcome measures (percentages of normal, abnormal or dead embryos) and (1) parental age and (2) date of experiment. For these analyses, each clutch (biological replicate) was treated as a single data point (*N*=36 clutches total). Coefficients of determination (*R*²), regression slopes and *P*-values testing whether slopes differed from zero were calculated using ordinary least squares regression in Microsoft Excel.

## Supplementary Material

10.1242/dmm.052632_sup1 Supplementary information

Table S1. Sources of variability in DTG toxicity.
