# Supplementary material for: Dolutegravir developmental toxicity is mitigated by magnesium and folate in zebrafish embryos
Source: Dis Model Mech. 2026 May 26;19(5):dmm052632. doi: 10.1242/dmm.052632 (PMC13267766; doi:10.1242/dmm.052632)
Supplement: Supplementary information [file dmm-19-052632-s1.pdf]

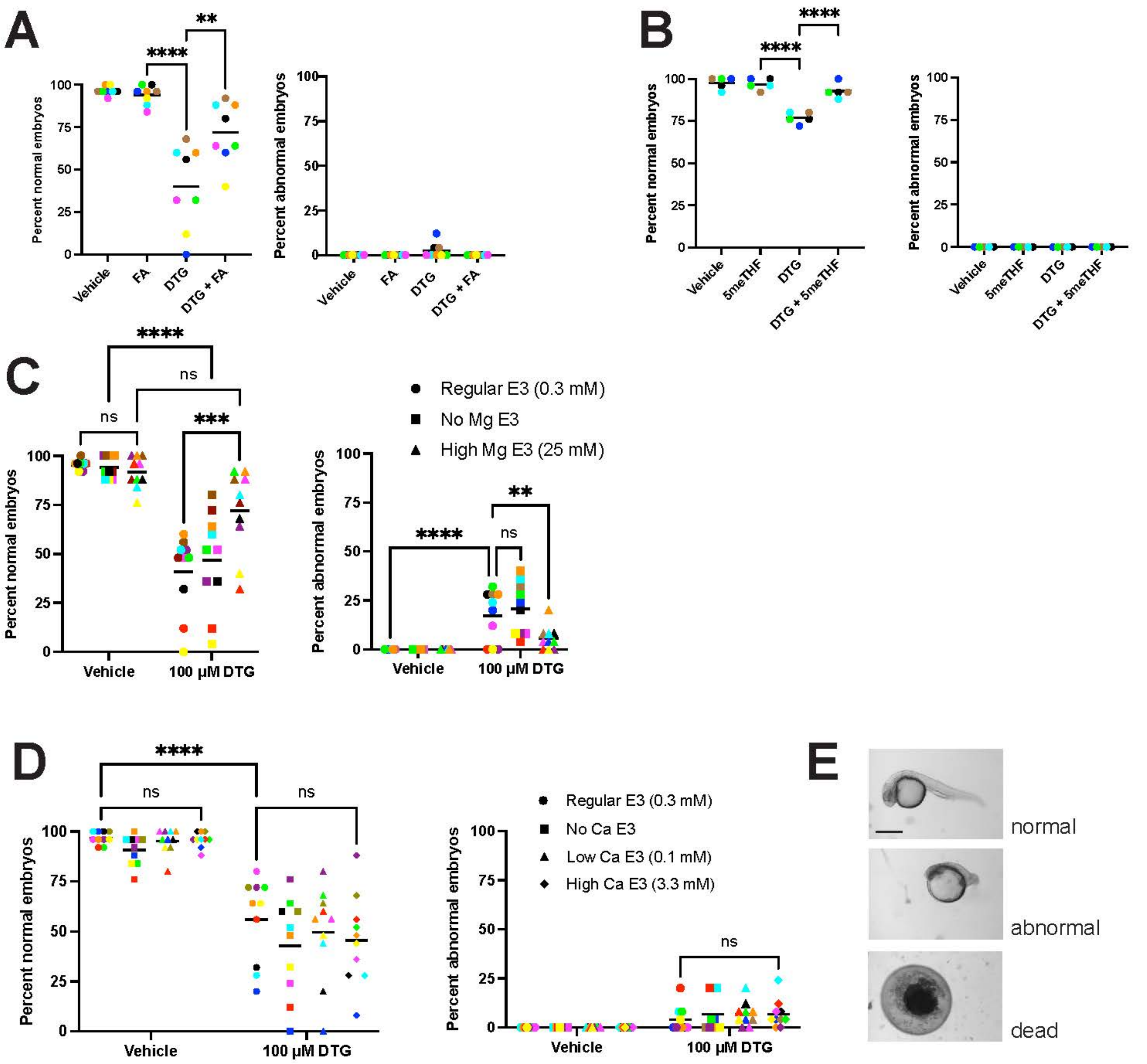

Fig. S1. DTG toxicity is rescued by folates and magnesium but not calcium. Same data as Figure 1, but here showing percent normal and abnormal embryos per clutch, where Figure 1 shows percent dead embryos. (A) Wild-type zebrafish embryos exposed to 100 µM dolutegravir (DTG) beginning at 2-4 hpf displayed reduced percent of normal embryos at 1 dpf, but no statistically significant difference in number of abnormal embryos, because most embryos were either normal or dead (see Figure 1). Co-treatment with folic acid (FA, 600 ng/mL) or (B) 5-methyltetrahydrofolate (5meTHF, 6 µg/mL) rescued the percent of normal embryos. One-way ANOVA with Tukey's multiple comparisons test. A, N=8 biological replicates (clutches), n=800 embryos total. B, N=5 replicates, n=500 embryos total. (C) Magnesium (Mg) supplementation (25 mM) rescued developmental toxicity of DTG-exposed embryos. N=10 replicates, n=1500 embryos total. (D) Calcium (Ca) had no protective effect. N=10 replicates, n=2000 embryos total. C, D, Two-way ANOVA with Tukey's multiple comparisons test. (E) Representative images of normal, abnormal, and dead embryos at 1 dpf. Lateral views, anterior to the left, scale bar 500 µm. For all graphs, each data point represents mean percent normal or abnormal embryos from a single clutch of 25 embryos (each clutch of embryos was produced from different parents). Within graphs in the same figure panel, data points of the same color are from the same clutch. **, $p<0.002$; ***, $p<0.0002$; ****, $p<0.0001$; ns, not significant.

Table S1. Sources of variability in DTG toxicity.

Available for download at
https://journals.biologists.com/dmm/article-lookup/doi/10.1242/dmm.052632#supplementary-data

Disease Models & Mechanisms • Supplementary information
